# West Nile Virus–infected Mosquitoes, Louisiana, 2002

**DOI:** 10.3201/eid1109.040443

**Published:** 2005-09

**Authors:** Marvin S. Godsey, Roger Nasci, Harry M. Savage, Stephen Aspen, Raymond King, Ann M. Powers, Kristen Burkhalter, Leah Colton, Dawn Charnetzky, Sarah Lasater, Viki Taylor, Charles T. Palmisano

**Affiliations:** * Centers for Disease Control and Prevention, Fort Collins, Colorado, USA;; † St. Tammany Parish Mosquito Abatement District, Slidell, Louisiana, USA

**Keywords:** West Nile virus, mosquitoes, Louisiana, *Culex quinquefasciatus*, research

## Abstract

Human cases of West Nile virus (WNV) disease appeared in St. Tammany and Tangipahoa Parishes in southeastern Louisiana in June 2002. Cases peaked during July, then rapidly declined. We conducted mosquito collections from August 3 to August 15 at residences of patients with confirmed and suspected WNV disease to estimate species composition, relative abundance, and WNV infection rates. A total of 31,215 mosquitoes representing 25 species were collected by using primarily gravid traps and CO_2_-baited light traps. Mosquitoes containing WNV RNA were obtained from 5 of 11 confirmed case sites and from 1 of 3 sites with non-WNV disease. WNV RNA was detected in 9 mosquito pools, including 7 *Culex quinquefasciatus*, 1 *Cx*. *salinarius*, and 1 *Coquillettidia perturbans*. Mosquito infection rates among sites ranged from 0.8/1,000 to 10.9/1,000. Results suggest that *Cx*. *quinquefasciatus* was the primary epizootic/epidemic vector, with other species possibly playing a secondary role.

## Abstract 2

*Culex quinquefasciatus* was identified as probable vector.

Since the first appearance of West Nile virus (WNV) (family *Flaviviridae*: genus *Flavivirus*) in the Western Hemisphere in 1999 (*1*), the virus has spread rapidly south and west from its initial focus in the New York City metropolitan area. By the end of 2001, WNV-infected mosquitoes, birds, horses, or humans had been reported from 27 states, and human cases of WNV disease occurred as far south as southern Florida and as far west as Arkansas and Louisiana (*2**,**3*).

In the northeastern United States, the primary epizootic/epidemic vector of WNV is *Culex pipiens*, a species that feeds primarily on birds (*4**–**6*). Other potentially important vector species, based on frequency of isolations of WNV or laboratory vector competence studies, include *Cx*. *restuans* and *Cx*. *salinarius* (*7**,**8*). WNV has been isolated from an additional 57 species, but their status as vectors is unknown (Centers for Disease Control and Prevention [CDC], http://www.cdc.gov/ncidod/dvbid/westnile/mosquitoSpecies.htm). In the southern United States, WNV was isolated from *Cx*. *quinquefasciatus*, *Cx*. *salinarius*, and *Cx*. *nigripalpus* in Florida and Georgia (*9*), *Cx*. *nigripalpus* in northern Florida (*10*), and from *Anopheles atropos*, *Deinocerites cancer*, and *Aedes taeniorhynchus* in the Florida Keys (*11*). However, the role these species play in epidemics of WNV disease in the southern states has not been determined. *Ae*. *albopictus* is common in urban, suburban, and rural residential settings throughout the southern states and is a competent laboratory vector of WNV (*12**,**13*). Although the virus has been isolated from *Ae*. *albopictus* in the Northeast (*14*), this species' importance in transmission of WNV to humans is unknown.

During May and June 2002, WNV infection was identified in chickens, horses, dead wild birds, and in pools of *Cx*. *quinquefasciatus* mosquitoes from St. Tammany Parish, on the north shore of Lake Pontchartrain in southeastern Louisiana (*15*). Human cases of WNV neuroinvasive disease began to appear in late June, and 27 cases were reported by the end of July. Intense local WNV transmission was indicated by the St. Tammany Parish Mosquito Abatement District's surveillance program, which detected WNV immunoglobulin (Ig)M antibody in 17% of their sentinel chickens and WNV antigen from 11 mosquito pools by the end of July (*15*). The human cases tended to cluster in 2 areas of St. Tammany Parish, Slidell and the Covington-Mandeville area. In neighboring Tangipahoa Parish, human cases were also being reported, with most clustering in the Hammond-Pontchatula area (Louisiana Department of Health and Hospitals, unpub. data).

The recognition of a growing outbreak of WNV disease in humans provided an opportunity to describe the transmission dynamics of WNV in locally occurring mosquitoes during epidemic transmission and to compare these dynamics to patterns seen in the northeastern states (*4**–**6*). Accordingly, we conducted an entomologic survey in St. Tammany and Tangipahoa Parishes during August 2002. The specific aims of the survey were to document species composition, relative abundance, and WNV infection rates in mosquitoes at residences of patients with confirmed cases and at residences of patients with suspected cases of WNV fever, the most likely locations where transmission to humans occurred. We were particularly interested in attempting to ascertain the importance of *Cx*. *quinquefasciatus* and *Ae*. *albopictus* as vectors of WNV in this epidemic.

## Materials and Methods

### Study Sites and Specimen Collection

Mosquitoes were collected in St. Tammany and Tangipahoa Parishes from August 3 to August 15, 2002. Two study sites were selected in each parish (denoted as St. Tammany A and B, and Tangipahoa A and B). These sites were located at or near residences of patients with confirmed cases of WNV neuroinvasive disease. As suspected cases of WNV fever (persons reporting as outpatients with undifferentiated febrile illness with headache) were identified, collections were made at the residences of these patients.

Mosquitoes were collected primarily by using CDC miniature light traps baited with dry ice to collect host-seeking females, Reiter gravid traps (*16*) to collect females seeking a location to deposit eggs, and ovitraps to collect eggs from container-breeding mosquitoes. Both light and gravid traps at the 4 initial study sites were operated for 24 h/day in an attempt to maximize the collection of *Ae*. *albopictus*, a daytime feeder. Some additional collections were made by using Fay-Prince traps and duplex cone traps and by aspirating resting adult mosquitoes from the outside of residences or other structures. Collections were transferred to 2.0-mL cryovials and frozen on dry ice until returned to the CDC laboratory in Fort Collins, Colorado, where they were stored at –80°C. Mosquito eggs collected in ovitraps were hatched in the insectary, reared to adulthood, held for 48 h at 27°C and 80% relative humidity, then identified and processed for virus testing as described below.

### Mosquito Processing and Testing

Mosquitoes were identified to species on a refrigerated chill table. Pools of <50 specimens sorted by species and collection site and date were triturated in 1.75 mL of diluent by using a Mixer Mill apparatus (Qiagen Inc., Valencia, CA, USA) and centrifuged (*17*). Supernatants from the mosquito suspensions were tested for the presence of WNV RNA by TaqMan reverse transcription–polymerase chain reaction (RT-PCR), and positive pools were retested by using a different primer set to confirm the presence of WNV RNA (*18*). Mosquito infection rates were determined by calculating the maximum likelihood estimate (MLE) with 95% confidence intervals (*19*).

## Results

### Mosquito Collections

Collections were made at 14 sites, 12 in St. Tammany Parish and 2 in Tangipahoa Parish. Residences of WNV neuroinvasive disease or fever case-patients are denoted by upper case letters. Non-case-patient residences are denoted by italicized lower case letters. Eight St. Tammany sites (A, C, D, E, F, *g*, I, J) were in or near the city of Slidell in the southeast corner of the parish, St. Tammany site B was located in Abita Springs, east of Covington, and 3 sites (K, *l*, *m*) were in Pearl River in the east-central region of the parish. The 2 Tangipahoa parish sites (A, B) were on the northwest and northern outskirts of Ponchatoula.

Trapping effort at each site and elapsed time between onset of illness and mosquito collection are shown in Table 1. Although traps were run for 24 h/day at some sites, only mosquitoes collected overnight are used to calculate mosquitoes per trap night. The earliest date of onset was June 21, and the latest date of onset was August 4. Mosquito collection dates ranged from 8 to 50 days after onset of illness. Trapping effort per site ranged from 2 to 60 trap nights for light trap collections, and from 2 to 59 trap nights for gravid trap collections. No notable changes in the weather occurred during the collection period that might bias comparisons of mosquito abundance.

**Table 1:** Trapping effort at West Nile virus (WNV) case-patient and non–case-patient residences, August 3–15, 2002

Parish	Site*	Case onset date	Collection dates	No. trap nights†		
				Light	Gravid	Other methods‡
St. Tammany	A	Jul 11	Aug 3–6	55	59	
	B	Jul 13	Aug 3–6	59	51	
	C	Jul 24	Aug 12–15	12	12	A
	D	Jul 29	Aug 12–15	8	8	A
	E	Aug 2	Aug 12–15	11	16	4 F, 4 D, A
	F	Aug 4	Aug 12–15	19	21	
	I	Jul 28	Aug 12–15	12	12	A
	J	Aug 4	Aug 12–15	3	3	
	K	Jul 29	Aug 13–15	6	6	
	*g*	Not given	Aug 13–15	9	15	3 F
	*l*	Not given	Aug 14–15	2	2	
	*m*	Not given	Aug 14–15	4	4	
Tangipahoa	A	Jul 11	Aug 7–10	54	59	
	B	Jun 21	Aug 7–10	60	48	

*Capital letters denote confirmed WNV neuroinvasive disease and WNV fever case sites; italicized lower case letters denote non-WNV case sites. †Only night collections used for trap night calculations. ‡A, aspiration outside buildings; F, Fay-Prince trap; D, duplex cone. Numbers denote trap nights; aspiration times not recorded.

A total of 31,215 mosquitoes were collected during the trapping period of August 3 to August 15 (Table 2). *Cx*. *erraticus* was the most commonly collected species, accounting for 28% of the total collected. *Cx*. *quinquefasciatus*, *Ae*. *albopictus*, *Coquillettidia perturbans*, and *Cx*. *salinarius* were other commonly collected species. Ovitraps yielded 335 *Ae*. *albopictus* and 778 *Ae*. *triseriatus*/*hendersoni* reared to adults. Aspirator collections yielded 658 mosquitoes of 16 species, of which 474 were *Ae*. *albopictus*. Cone traps collected 33 mosquitoes (9 species) and Fay-Prince traps yielded 214 mosquitoes (15 species). Mosquitoes were sorted into 2,471 pools for processing and virus testing.

**Table 2:** Mosquito species collected in St. Tammany and Tangipahoa Parishes, Louisiana, August 3–15, 2002

Species	No. of mosquitoes				
	No. of pools	Light traps	Gravid traps	Other methods*	Total (%)
*Culex erraticus*	310	8,319	411	3	8,733 (27)
*Cx*. *quinquefasciatus*	311	539	6,326	98	6,963 (22)
*Aedes albopictus*	321	1,007	1,457	860	3,324 (11)
*Coquillettidia perturbans*	107	2,159	114	0	2,273 (7)
*Cx*. *salinarius*	144	1,809	155	49	2,013 (7)
*Culex* species	171	389	1,318	37	1,744 (6)
*Ae*. *triseriatus/hendersoni*†	159	198	86	782	1,066 (3)
*Psorophora ferox*	117	909	27	37	973 (3)
*Ps*. *howardii*	112	680	2	4	686 (2)
*Uranotaenia sapphirina*	59	631	48	0	679 (2)
*Ae*. *vexans*	83	500	18	24	542 (2)
*Ae*. *infirmatus*	90	465	7	17	489 (2)
*Ae*. *atlanticus/tormentor*†	78	371	28	37	436 (1)
*Ae*. *taeniorhynchus*	41	231	12	46	289 (<1)
*Aedes* species	59	198	15	17	230 (<1)
*Ps*. *columbiae*	54	188	4	4	196 (<1)
*Anopheles crucians* complex	66	184	4	3	191 (<1)
10 other species	193	317	65	6	388 (1)
Total	2,471	19,094	10,097	2,024	31,215 (100)

*Other methods: mechanical aspirator, duplex cone trap, Fay-Prince trap, oviposition trap. †Not identified to species.

Relative population densities (light trap or gravid trap counts per trap night) of the species in which we detected WNV RNA, and of *Ae*. *albopictus*, were calculated for case and non-case sites (Table 3). For most species, light trap counts per night greatly exceeded gravid trap counts. For *Cx*. *quinquefasciatus*, however, gravid trap counts were 7–58 times greater than were light trap collection counts. Neither gravid traps nor light traps collected large numbers of *Ae*. *albopictus*. Light trap counts per trap night for *Ae*. *albopictus* were approximately the same as gravid trap counts except at site *l* where 35.5 mosquitoes were collected per gravid trap night compared to 4.5 per light trap night.

**Table 3:** Population densities of selected mosquito species at West Nile virus (WNV) case-patient and non–case-patient residences*

	No. of mosquitoes collected per trap night (LT/GT)†			
	*Culex quinquefasciatus*	*Cx*. *salinarius*	*Aedes albopictus*	*Coquillettidia perturbans*
St. Tammany				
A	1.7/21.7	1.6/0.05	1.8/1.2	0.2/0
B	0.5/3.7	1.5/0.1	0.9/1.7	0/0
C	0.3/17.4	2.6/0	1.5/1.0	0/0
D	0.6/18.1	1.0/0	1.6/2.8	0/0
E	3.7/44.1	5.4/0.06	2.2/3.0	0/0
F	1.0/19.4	5.0/0	2.8/2.1	0/0
I	1.3/15.7	3.2/0	2.4/2.9	0/0
J	2.3/39.3	6.7/0.3	2.3/0.3	0/0
K	1.5/12.2	1.0/0	9.8/11.2	0.2/0
*g*	4.4/59.6	6.7/0.07	4.1/3.6	0/0
*l*	6.5/105.0	0.5/0	4.5/35.5	0.5/0
*m*	7.0/142.8	0/0	0.8/0.3	0/0
Tangipahoa				
A	0.02/0.4	6.4/1.0	0.7/1.9	17.7/1.6
B	3.2/15.1	8.0/1.2	5.2/8.5	16.2/0.3

*Capital letters denote confirmed WNV neuroinvasive disease and WNV fever case sites; italicized lower case letters denote non-WNV case sites. †LT, light trap; GT, gravid trap; only night collections used for trap night calculations.

No relationship was shown between the population densities of the species examined and whether the site was a case-patient or non–case-patient residence, except for *Cx*. *quinquefasciatus*, for which much higher densities were found at sites of non-case-patients. *Cx*. *quinquefasciatus* gravid trap counts per trap night ranged from 0.4 to 44.1 for confirmed WNV disease case-patient residence sites, and 59.6 to 142.8 for non–case-patient sites (p<0.001, Wilcoxon rank sum test).

### WNV Detection

WNV RNA was detected in 9 mosquito pools by TaqMan RT-PCR (Table 4). Five viral RNA positive pools were from St. Tammany Parish and 4 were from Tangipahoa. Seven of the positive pools contained *Cx*. *quinquefasciatus*; 4 of these were from St. Tammany Parish, and 3 were from Tangipahoa. The other 2 positive pools consisted of a pool of *Cx*. *salinarius* from St. Tammany and a pool of *Cq*. *perturbans* from Tangipahoa. All of the WNV-positive *Cx*. *quinquefasciatus* were collected in gravid traps, while the positive *Cx*. *salinarius* and *Cq*. *perturbans* were collected in light traps. No virus was detected in mosquitoes collected by the other methods. WNV infection rates ranged from 0.81/1,000 to 10.91/1,000 by MLE (Table 4). The highest infection rate was seen in *Cx*. *salinarius* and the lowest in *Cq*. *perturbans*. Infection rates in *Cx*. *quinquefasciatus* were similar among sites (2.31/1,000–5.64/1,000).

**Table 4:** Estimated mosquito pool West Nile virus (WNV) infection rates per 1,000 mosquitoes and 95% confidence intervals (CIs)*

Parish	Site†	Sampling period	Trap type‡	WNV+ pools	Species	No. mosquitoes tested	Infection rate: MLE (95% CI)
St. Tammany	B	Aug 3–6	Light	1	*Cx*. *salinarius*	92	10.91 (5.46–21.83)
	E	Aug 12–15	Gravid	2	*Cx*. *quinquefasciatus*	829	2.61 (1.31–5.22)
	F	Aug 12–15	Gravid	1	*Cx*. *quinquefasciatus*	427	2.31 (1.16–4.62)
	*l*	Aug 14–15	Gravid	1	*Cx*. *quinquefasciatus*	223	5.64 (2.82–11.28)
Tangipahoa	A	Aug 7–10	Light	1	*Cq*. *perturbans*	1,223	0.81 (0.41–1.62)
	B	Aug 7–10	Gravid	3	*Cx*. *quinquefasciatus*	922	3.41 (1.71–6.82)

*Calculated by using a bias-corrected maximum likelihood estimate (MLE). †Capital letters denote confirmed WNV neuroinvasive disease and WNV fever case sites; italicized lower case letters denote non-WNV case sites. ‡Light denotes CO_2_-baited CDC miniature light trap; gravid denotes Reiter gravid trap.

No relationship was found between the relative densities of mosquitoes collected and the finding of WNV-infected mosquitoes (Tables 3 and 4). Three infected pools of *Cx*. *quinquefasciatus* were collected from Tangipahoa site B, with 15.1 mosquitoes per gravid trap night, whereas no infected pools were collected from St. Tammany site *m*, which had the highest *Cx*. *quinquefasciatus* count per gravid trap night (142.8). Likewise, the only WNV-infected *Cx*. *salinarius* pool was from St. Tammany site B, which had 1.6 mosquitoes per light trap night, 1 of the lower density sites for that species. Eight other sites had higher light trap counts but no WNV-positive mosquitoes were detected. *Cq*. *perturbans* was found in high densities at only Tangipahoa sites A and B, and the densities at these sites were similar at 17.7 and 16.2 per light trap night, respectively. Infected *Cq*. *perturbans* were found only at Tangipahoa site A.

Detection of WNV-infected mosquitoes was not influenced by elapsed time between dates of onset of illness (a surrogate for date of infection) and mosquito collection dates. We obtained 3 isolates from Tangipahoa site B, where the date of onset was 47–50 days before mosquito collection (Tables 1 and 4).

## Discussion

The results of our survey indicate that the natural history of WNV in the southern United States is similar to that seen in the northern states, where *Cx*. mosquitoes, especially *Cx*. *pipiens*, *Cx*. *restuans*, and *Cx*. *salinarius*, are thought to be the species primarily involved in enzootic, epizootic, and epidemic transmission (*3**–**6*). Seven of 9 (78%) WNV-infected mosquito pools were *Cx*. *quinquefasciatus*. Both *Cx*. *pipiens* and *Cx*. *quinquefasciatus* are primarily ornithophilic, although some studies indicate that *Cx*. *quinquefasciatus* feeds more readily on mammals (*20**–**22*). One of the 2 other positive pools was of *Cx*. *salinarius*, which feeds primarily on mammals (*20**–**22*). WNV has been isolated frequently from this species (*5**,**6**,**23*), and laboratory studies indicate that it is a competent vector (*8*). *Cx*. *salinarius* has been associated with an outbreak of human WNV illness in New York City (*6*) and appears likely to be important in transmitting WNV to humans and domestic mammals in the southern United States as well. The other positive pool was of *Cq*. *perturbans*. WNV isolates previously have been obtained from this species, but it is an inefficient vector in the laboratory (*8*).

Eight mosquito pools containing WNV RNA were collected at 5 (45%) of 11 confirmed WNV case-patient residences, while the remaining pool was from 1 (33%) of 3 non–case-patient sites. This finding suggests that many, perhaps most, human infections are acquired near their residences.

Although substantial numbers of *Ae*. *albopictus* were tested, no virus was detected in this competent laboratory vector of WNV. This finding was perhaps due to the blood-feeding habits of this species. Two studies of engorged specimens wild caught in the continental United States found that 1% and 17% of blood meals were taken from birds (*24**,**25*). The remaining meals were from a variety of mammals, including humans. In our study area, relatively few blood meals may have been taken from birds, thus reducing the exposure of host-seeking *Ae*. *albopictus* to the high-titered levels of WNV viremia seen in many species of birds. Little data have been published on WNV viremia levels in mammals, but in horses, dogs, and cats, viremia levels are transient, of low titers, or both (*12**,**26*). If this condition is also the case for other mammalian species, then most blood meals taken by *Ae*. *albopictus* from WNV-infected hosts would be below the threshold titer necessary to initiate infection.

In our study, gravid traps were clearly preferable to light traps as an effective surveillance tool for detecting WNV RNA in mosquitoes. All the positive *Cx*. *quinquefasciatus* pools and 91% of total *Cx*. *quinquefasciatus* were from gravid traps. The other 2 WNV-positive pools were from mosquitoes collected in light traps. Gravid traps were a more effective means of collecting *Ae*. *albopictus* than were light traps. Unlike *Cx*. *quinquefasciatus*, most female *Ae*. *albopictus* collected in gravid traps were not gravid, and numerous males were also collected. *Ae*. *albopictus* were also readily collected by aspiration and ovitrapping.

Although active transmission of WNV was still occurring at the time of our collection efforts during the first half of August, most human patients had dates of onset between late June and late July. Thus, the relative numbers and species composition we observed may not have been representative of the situation when most human infections were occurring. Mosquito control activities intensified in St. Tammany Parish in response to the high level of WNV activity (*15*). Mosquito surveillance by the parish showed large reductions in total mosquito counts and in *Cx*. *quinquefasciatus* counts in CDC light traps and in New Jersey light traps from May to August. Eleven WNV antigen-positive mosquito pools were detected, all in June and July. Ten of these positive pools were of *Cx*. *quinquefasciatus*, and 1 was of *Cx*. *salinarius*, similar to our findings in August. Notably, the number of sentinel chickens developing WNV IgM antibody peaked during the third week of July, declined during early August, then rose again during late August (*15*). This finding suggests that exposure of sentinel chickens to infected mosquitoes was ongoing, and perhaps increasing, during the period of our study. Serologic conversions in sentinel chickens continued to be detected into November. Serologic studies of wild birds caught in mist nets in St. Tammany Parish were conducted in August, and again in October (*27*). These data indicated that enzootic WNV transmission continued to occur in the parish, although likely at a reduced level, after human cases were no longer being reported. Long-term studies are needed to monitor the transmission dynamics of WNV in mosquito populations during epidemic and nonepidemic years.
